# Wiskott Aldrich syndrome: healthcare utilizations and disparities in transplant care

**DOI:** 10.1038/s41598-021-84328-0

**Published:** 2021-02-25

**Authors:** Nikki Agarwal, Divyaswathi Citla Sridhar, Sindhoosha Malay, Nirav Patil, Anjali Shekar, Sanjay Ahuja, Jignesh Dalal

**Affiliations:** 1 Cleveland Clinic, 9500 Euclid Avenue, Cleveland, OH 44195 USA; 2 Rainbow Babies and Children Hospital, Case Western Reserve University, 11100 Euclid Avenue, Cleveland, OH 44106 USA; 3 School of Medicine, Case Western Reserve University, 1200 Wolstein Research Building, Cleveland, OH 44106 USA; 4 University Hospitals Cleveland Medical Center, 1200 Wolstein Research Building, Cleveland, OH 44106 USA

**Keywords:** Health care, Medical research

## Abstract

Wiskott Aldrich syndrome (WAS) is a rare disease and hematopoietic stem cell transplant (HCT) is considered the treatment modality of choice for WAS. We conducted a cross-sectional analysis on the KIDS’ pediatric inpatient database and compared hospitalization rates, complications and healthcare utilizations in the transplant and non-transplant arms. Of the 383 pediatric admissions with diagnosis of WAS between 2006–2012, 114 underwent transplant and 269 did not. The non-transplant arm included older children, female patients and more African Americans. Death rates, income and payer source were similar in both arms, however the total charge for each admission was higher in the transplant arm. Emergency room visits were similar but non-elective admissions were more in the non-transplant arm. Length of stay was prolonged in the transplant arm. When comparing morbidities, lymphomas, ulcerative colitis and autoimmune complications of WAS were seen only in the non-transplant arm. Our study shows that transplant is the largest contributor to healthcare utilization in WAS patients. We identified healthcare disparities based on race and socioeconomic status and found that this rare disease is being appropriately directed to centers with HCT expertise. We noted a change in practice moving away from splenectomy in WAS patients.

## Introduction

Wiskott Aldrich syndrome (WAS) is a rare X-linked disorder characterized by eczema, small platelets with thrombocytopenia and combined immunodeficiency with increased risk for autoimmune disorders and cancers^1,2^. The incidence is 1 to 10 per million live male births, with average age at diagnosis being 24 months in families without a previously affected family member^3^. WAS has a wide range of phenotypes depending on the expression of the WASP gene. WAS protein is a 502 amino acid multidomain protein encoded by twelve exons on the X-chromosome and is exclusively expressed in hematopoietic cells. There is a strong correlation between the level of WAS protein in the cells and the severity of WAS phenotype. Complete absence of protein results in severe functional defect in multiple hematopoietic cell lineages leading to thrombocytopenia, lymphopenia and abnormal lymphoid and myeloid functions^4^. Milder form of the disease includes X-linked thrombocytopenia (XLT), where WAS protein expression is present in less than normal levels. This presents with thrombocytopenia, mild eczema and mild immunodeficiency. Activating mutation in GTPase binding domain of WAS protein can present with X-linked neutropenia (XLN), which manifests as neutropenia and variable myelodysplasia^2^. A scoring system has been developed to identify these distinct clinical phenotypes caused by mutation in the WASP gene^5^. Milder forms are managed conservatively, however patients with severe forms of WAS develop complications such as severe infections, autoimmunity or malignancy; tend to do poorly and generally die in early/mid adulthood. Therapeutic options include supportive therapy such as prophylactic IVIG infusions, antibiotic therapy, immunosuppression and splenectomy. The only curative therapies, especially for the severe forms are allogeneic bone marrow transplant and gene therapy^6–8^. Hematopoietic stem cell transplant (HCT) as a treatment modality for WAS was first employed in 1968^3^. Since then, use of matched sibling donor for HCT has become a standard of care for WAS^6–8^.

As with rare diseases, there is a paucity of randomized control trials in WAS. Observational studies have been conducted to define disease events and recognize outcomes of WAS patients treated with different therapeutic modalities. Randomized studies have not been done to compare outcomes with transplant versus supportive care, given the natural history of the disease.

There are no studies with a direct comparison of complications and health care utilization in patients who underwent transplant versus no transplant. This was considered an important question given that transplant is considered standard of care especially for the severe phenotypes. We conducted a cross sectional analysis on a large pediatric inpatient database to compare the complications and health care utilization in pediatric patients with WAS who underwent HCT versus those who received only supportive care.

## Methods

### Study design and sample

This was a cross-sectional analysis of pediatric hospitalizations (age < 21 years) of patients with WAS using the Kids’ Inpatient Database (KID), prepared by the Agency for Healthcare Research and Quality (AHRQ) as part of the Healthcare Resource Utilization Project (HCUP)^9–11^. The KID database is a pediatric all-payer inpatient database of pediatric discharges at children’s hospitals, academic centers and community hospitals from 47 participating states. Since this study involves analysis of a publicly available de-identified data, it was exempt from Institutional Review Board review.

### Outcomes

The primary outcome was to measure hospitalization among patients with WAS, who underwent HCT versus patients who received supportive care. Our secondary outcomes were to compare incidence of WAS associated complications among patients who did and did not receive HCT and compare health care utilization among patients in both groups.

### Clinical variables

Hospitalized patients with WAS were identified with an *International Classification of Disease – ninth revision* (ICD-9) code of 279.12. Among these patients, those who underwent HCT were identified using ICD-9 diagnosis code of V4281 and DRG version 24 of 481. Data collected included patient age, sex, race, death during hospitalization, length of stay in days, elective admission, number of procedures, total charges for the admission, location of the patient and primary insurance status (Medicare, Medicaid, Private Insurance and Self-pay). All ICD-9 codes listed under diagnosis were reviewed to identify complications associated with WAS and complications associated with HCT.

### Statistical analysis

HCUP-NIS sampling design and analysis have been described in previous HCUP publications^12,13^. We used the standard published algorithm and applied sampling weights to generate nationwide estimates. Results mentioned in this paper are weighted nationwide estimates. Data was presented as mean (standard deviation) or frequency (percentage) as appropriate. Continuous data was compared with the two-tailed independent sample t-test while categorical data was compared with the Rao-Scott adjustment for chi square test. All results are two-tailed and at the 95% confidence level. P-value of less than 0.05 was considered statistically significant for the analysis. All the analysis was performed using SAS software, version 9.4 (SAS Institute, Cary, NC). HCUP methods series recommendations were followed for estimates for standard errors and confidence intervals^10^.

## Results

### Descriptive statistics (Table 1)

**Table 1:** Demographic characteristics of wiskott-aldrich syndrome patients.

	Overall (N = 383)	WAS observations		P value
		no HCT (n = 269)	HCT (n = 114)	
Age in years, mean ± SD	5.03 ± 0.73	5.63 ± 0.90	3.60 ± 0.56	0.0564 ^a^
Length of stay in days, mean ± SD	10.32 ± 1.25	6.88 ± 1.18	18.38 ± 1.99	< 0.001 ^a^
**Sex, n (%)**				–
Male (0)	374 (98.12)	260 (97.32)	114 (100.00)	
Female (1)	7 (1.88)	7 (2.67)	0 (0.00)	
**Age, n (%)**				0.0612^b^
0 to 5 years	240 (44.98)	153 (56.82)	88 (77.13)	
6 to 10 years	76 (15.37)	62 (22.97)	15 (12.83)	
10 to 15 years	35 (13.11)	25 (9.31)	10 (8.69)	
15 to 20 years	31 (14.18)	29 (10.91)	2 (1.35)	
**Race, n (%)**				0.4118^b^
White (1)	137 (43.43)	88 (32.87)	49 (42.84)	
Black (2)	57 (18.06)	49 (18.33)	8 (6.79)	
Hispanic (3)	79 (24.96)	56 (20.92)	23 (19.80)	
Others (4)	110 (28.67)	75 (27.87)	35 (30.56)	

HCT—hematopoietic stem cell transplantation, SD—standard deviation.

^a^Two-tailed independent sample t-test.

^b^Rao-Scott adjustment for chi-square test.

**Figure 1:**
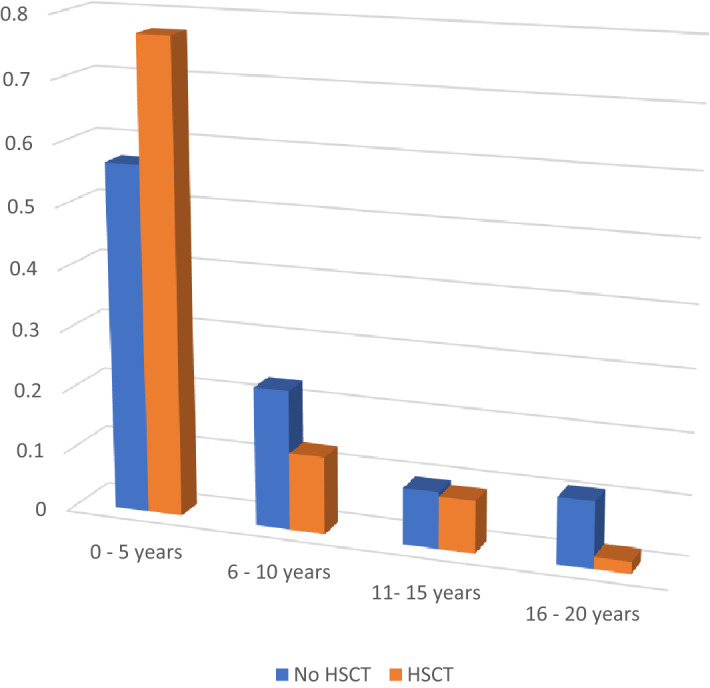
Age distribution—No HCT vs HCT.

We analyzed a total of 383 pediatric hospitalizations with the diagnosis of WAS. 114 had undergone HCT and 269 had not undergone HCT. Overall mean age of admission was 5 years, with admissions in transplant arm having a mean age of 3.6 years compared to 5.63 years for non-transplant arm. When subcategorized for age (Fig. 1), most admissions were in the 0–5 year age in both groups, however there were more admissions in the non-transplant arm after 6 years of age compared to transplant arm (p = 0.0612). There were 7 female patients in the study group and all of them were in the non-transplant arm, the rest of admissions were males. The phenotype of these 7 females could not be derived from the database, and it was not possible to separately identify the cause of their admissions due to database limitations. There were more African Americans(18.3%) in the non- transplant arm compared to transplant arm (6.8%). 50% patients overall had Medicaid with equal distribution in both arms, the rest had either private insurance or not reported (p = 0.395).

### Inpatient mortality

Death rates during hospitalization were comparable in both arms (1.61% in the non-transplant vs 1.15% in the transplant arm). Due to the limitations in acquiring data from a dataset, we were not able to discern the individual causes of death.

### Health care utilization

Although payer source and median household income were not significantly different between the transplant and the non-transplant arms, the total charge for each admission was higher in the transplant arm. (Table 2). Emergency room visits were similar in both arms (p = 0.56). Elective admissions were more in the transplant arm compared to the non-transplant arm (p = 0.0244) as expected. Length of stay was prolonged in transplant arm (18 days) compared to non-transplant arm (6.8 days) and was statistically significant (p < 0.001). The frequency of a major operating room procedure was higher in the transplant arm (38.65%) when compared to the non-transplant arm (6.05%) (p < 0.001). These procedures were not individually identifiable in the dataset. Comparing the morbidities, lymphomas, ulcerative colitis and autoimmune complications of WAS were seen only in the non-transplant arm. Autoimmune complications included autoimmune cytopenias, hemolytic anemias, vasculitis, hypersensitivity angiitis and rarely connective tissue disorders. Patients with WAS were hospitalized in 85 centers across US, among these centers, only 10% centers (9/85) had 42% (2.8–9.1% each) of the hospitalizations and 90% of the centers had less than 2.5% of hospitalizations each (Table 3).

**Table 2:** Hospitalization characteristics of Wiskott-Aldrich syndrome patients in HCUP kids database.

	Overall (N = 383)	WAS Observations		p-value
		no HCT(n = 269)	HCT(n = 114)	
Number of procedures per admission, mean ± SD^c^	2.27 ± 0.21	1.89 ± 0.29	3.18 ± 0.33	**0.0094**^**a**^
**Number of procedures per admission, n (%)**				**0.0031**^**b**^
None	137 (35.73)	103 (38.33)	34 (29.57)	
1	69 (18.17)	55 (20.35)	15 (13.01)	
2	58 (15.15)	48 (17.70)	10 (9.13)	
≥ 3	118 (30.95)	63 (23.62)	55 (48.28)	
**Death during hospitalization, n (%)**				0.2528^b^
Did not die (0)	376 (98.38)	266 (98.85)	111 (97.28)	
Died (1)	6 (1.61)	3 (1.15)	3 (2.72)	
**Admission, n (%)**				**0.0244**^**b**^
Elective (1)	108 (28.28)	62 (23.26)	46 (40.06)	
Non-elective (0)	273 (71.72)	205 (76.74)	68 (59.94)	
**ED Visit, n (%)**				0.5619^b^
No (0)	249 (65.20)	178 (66.29)	71 (62.61)	
Yes (1)	133 (34.80)	91 (33.71)	42 (37.39)	
Major Operating room procedure^d^ per admission, n (%)	60 (15.74)	16 (6.05)	44 (38.65)	** < 0.001**^**b**^
Hospital total charges, mean ± SD	$140,925 ± $33,908	$87,093 ± $28,599	$2,67,381 ± $49,501	** < 0.0001**^**a**^
**Payer info, n (%)**				0.3950^b^
Medicare	0 (0.00)	0 (0.00)	0 (0.00)	
Medicaid	189 (49.52)	131 (48.81)	58 (51.19)	
Private insurance	163 (42.77)	120 (44.79)	43 (38.00)	
Others	29 (7.70)	17 (6.39)	12 (10.80)	

HCT – Hematopoietic stem cell transplantation, SD – Standard Deviation.

^a^Two-tailed independent sample t-test.

^b^Rao-Scott adjustment for chi-square test.

^c^Including line placement, lumbar punctures, bone marrow biopsies.

^d^Included line placements, bone marrow biopsies, other surgical procedures, endoscopies.

**Table 3:** Distribution of hospitalizations among different hospitals in the country.

Hospital ID	Total (N = 383)	Wiskott Aldrich syndrome patients	
		NO HCT (N = 269 )	HCT (N = 114)
39,023	35 (9.29)	11 (4.20)	24 (21.30)
20,461	34 (8.88)	19 (7.14)	15 (12.98)
6399	16 (4.21)	16 (5.99)	0 (0.00)
10,186	15 (3.80)	15 (5.41)	0 (0.00)
48,227	14 (3.62)	14 (5.14)	0 (0.00)
30,503	13 (3.47)	6 (2.20)	7 (6.49)
6370	13 (3.38)	13 (4.81)	0 (0.00)
6632	11 (2.86)	11 (4.07)	0 (0.00)
12,155	11 (2.95)	6 (1.69)	5 (4.26)
Others	220 (57.59)	158 (58.73)	63 (55.26)

76 Hospitals were categorized as Others as none of them had more than 7 patients (unweighted).

## Discussion

This study is one of the largest database reviews for pediatric patients with WAS, with data from all over the United States over a 6-year period. We found very interesting results with the review of the KIDS inpatient database made available through HCUP.

Our study showed 7 female patients with WAS seeking medical care, and all of them were in the non-transplant arm. We were not able to identify the causes for their admission through the dataset, however on further literature review, females with WASP gene mutations were seen to be usually asymptomatic as their mature blood cells have nonrandom X chromosome inactivation. Prchal et al^14^ looked at X-chromosome expression in the mother of two siblings with WAS and suggested that there is selection against cells expressing the defective WASP gene in the hematopoietic system in carriers. Fearon^15^ et al. studied eight carriers of WAS defect and identified skewed patterns of X chromosome inactivation in T-cells, granulocytes and B cells. Multiple other studies have been done to look at the carrier state in WAS^16–18^. Bryne et al. reported a case where the female patient had progressive thrombocytopenia requiring HCT at 15 months of age^19^. She was found to have skewed inactivation of the X chromosome inherited from her mother. Another case report for a female presenting with mild WAS symptoms was found to have 60% of normal WASP levels in peripheral blood mononuclear cells, compared to actual cases of WAS where WASP levels were 0–20%^20^. She was noted to have heterozygosity with 2 distinct populations in her lymphocytes and monocytes. Many other case reports have been described with females with WAS symptoms^21–25^. Hypothesis for disease expression in a female with heterozygosity to the WASP mutation are either : abnormality in transcriptional X chromosome silencing in the hematopoietic stem cells, or abnormality in the process that leads to preferential survival and expansion of cells that bear the active wild-type X chromosome^26^.

Post-transplant hospitalization is the largest contributor to health care utilization for transplant patients. We noted that the length of stay (LOS) per admission was longer in transplant patients compared to the non-transplant patients, especially in admissions associated with transplant as a procedure code, with mean LOS of 8 days for non-transplant arm and 18 days in the transplant arm. In a study using national claims database of commercially insured population in the United States, Majhail et al^27^ showed that the median duration of hospitalization was 19 days for autologous HCT recipients and 31 days in allogenic HCT recipients. The median days of hospital stay after the transplant was 21 days in < 20-year age group for autologous HCT and 42 days for allogenic HCT. In another multicenter cohort study^28^, LOS compared between different graft sources in the first 100 days after transplant for acute leukemias showed umbilical cord blood (UCB) and mismatched unrelated donor transplants (MMUD) to have a longer hospital stay compared to matched unrelated donors (MUD). The median value of LOS in the pediatric group were 50 days for single UCB, 54 days for double UCB and 60 days for MUD HCT. Our study suggests a much lesser LOS in WAS patients, but the graft source for our patients was not available in the database.

Due to restricted availability of HCT, concerns have been raised regarding disparities based on race, socioeconomic status, education and insurance status. Our study revealed more African Americans (18.3%) in the non-transplant arm compared to transplant arm (6.8%). This disparity could be explained by lack of donor sources, access to HCT and outcomes of HCT. Cancer facts and figures summarized by the American Cancer Society in 2019 show highest incidence of cancer and deaths in African Americans when compared to other races^29^. HCT is an expensive procedure, hence socioeconomic factors create a barrier to its easy access. Also, allogenic HCT needs appropriate HLA-matched donors, and the likelihood of matching between two random individuals increases if they are of the same race. The National Marrow Donor Program’s Registry^30^ has about 16 million potential donors, 7% of them are African Americans, 6% Asians and 1% American Indian/Alaskan Native, and they have ongoing initiatives to increase the diversity of the pool. Barker et al^31^ in a prospective study conducted between 2005–2017 found that increasing registry size has not resolved the racial disparity and suggested use of alternative graft sources such as cord blood transplant. Unavailable matched donors continue to be a barrier to HCT in these populations. Given the advances seen with haploidentical donor transplants, it will be interesting to see if this improves donor options in the ethnic groups not well represented in the donor databases.

WAS is a disease identified at a young age due to multiple infections or bleeding manifestations. Sullivan et al^32^ found that 36% patients with WAS experience non-HCT associated deaths at a mean age of 8 years, mostly due to infections (44%), bleeding (23%) and malignancy (26%). This study also found that the median survival was 20 years in patients with WAS with supportive care only. Our study showed most admissions were in the 0–5 year age group for both arms, which suggests early identification of the disease. Our hypothesis is due to increased awareness and ease of diagnosis, cases are more easily identified. Also, greater number of children > 5 years age required admission in the non-transplant arm, suggesting increased complications and medical needs with increased age. This indicates the increased healthcare utilization in the non-transplant arm in older age group. We were further able to consolidate this hypothesis by looking at the complications in both arms. We found that none of the patients in the transplant arm developed lymphomas, whereas 5.6% patients in the non-transplant arm developed lymphomas. Malignancies in untreated WAS has been reported as 13%^32^ and 22%^33^ in two different case series, including lymphomas, leukemias and myelodysplasia. Similarly, autoimmune manifestations are much more common in untreated WAS, ranging from 22 to 72% in various case series^32–35^. This was similarly reflected in our study where ulcerative colitis was noted only in the non-transplant arm compared to the transplant arm. Due to limitation of our database, we were unable to compare the course of WAS in the two arms beyond 20 years of age. It is important to note that this study focuses on inpatient hospitalizations in the pediatric age group. It is highly possible that the frequency of complications and therefore hospitalizations are much higher in the non-transplant arm in the adult population.

Our study did not show splenectomy done in either arm. There have been controversies regarding splenectomy if a potential donor is present, but this represents a shift in practice even in the non-transplant arm. Mullen et al^36^ had shown median survival of 25 years in splenectomized non HCT patients, compared to less than 5 years in unsplenectomized patients. Infectious complications related to splenectomy has been shown to be significant in WAS patients receiving HCT^37^. Moratto et al^38^ observed the long term outcomes for WAS and HCT over a 30 year period and showed that splenectomy may normalize platelet counts after HCT, however, there are associated risks of potentially fatal infections that need to be considered. Imai et al.^33^ had similar results of improvement in platelet counts after splenectomy, but with increased risk of life-threatening meningitis/sepsis. Our study shows a significant shift in practice away from splenectomy for management of thrombocytopenia.

Our study did not show significant difference in mortality between the two arms, but it is not reliable given that ours is an inpatient database. However, our study agrees with other studies that HCTs done at < 5-year age group have better outcomes^37,38^.

## Strengths and limitations

The KID inpatient database is one of the largest pediatric inpatient databases, with each data set providing 3 years of pediatric hospitalization data, with roughly 2 to 3 million discharges (unweighted), which corresponds to a national estimate of 6 to 7 million (weighted) discharges per data set. Our study has incorporated 2 datasets, which amounts to about 12–14 million (weighted) discharges and therefore, is a very powerful tool to study a rare disease like WAS.

There are several limitations to this study that need to be discussed. Since this is an administrative dataset, there are limitations to using it for secondary analysis of clinical outcomes. The data is based on each hospitalization and is not patient specific, hence we are unable to provide exact rates based on patient numbers. Since each hospitalization lists a set of diagnoses, the exact reason for hospitalization could not be identified. The causes of death in the inpatient hospitalizations could not be determined as well. This is an inpatient dataset, therefore outpatient care and mortality in the outpatient setting cannot be assessed. There might be errors secondary to incorrect ICD-9 coding, which are not avoidable in a large dataset study like these. We may have missed XLT and XLN as these are milder diseases and may have been billed under different ICD codes which we did not pick up during our statistical analysis. However, there have been several studies that have looked at the sensitivity of ICD-9 codes^12,13^.

We were unable to compare the healthcare utilization data for the transplanted patients before and after transplant due to the limitations in the database set, however that will be another interesting study to see if utilization decreased post-transplant. This dataset is a pediatric dataset for < 21 years of age, and hence we were not able to analyze the course of the disease beyond that age. Thus, our study may not reflect the total healthcare utilization spanning older age groups, especially in the non-transplant arm.

## Conclusions and future scope

In conclusion, our study identifies that HCT is the largest contributor to healthcare utilization in pediatric WAS patients. It also identifies the healthcare disparities based on race and socioeconomic status. There is a disparity in use of splenectomy in our database compared to other studies which suggest a change in practice in this immunodeficient population. We also found that there are centers across the nation which contribute to maximum inpatient admissions for WAS, noting that this rare disease is being appropriately directed to over 80 pediatric hospitals in 47 states where the expertise for HCT exist . There is a need for a bigger national database and longitudinal studies to follow these patients and assess for long term complications.

## Data Availability

KIDS database at www.hcup-us.ahrq.gov/kidoverview.jsp are available on request and with permission from HCUP.

## Abbreviations

WAS: Wiskott-Aldrich Syndrome
HCT: Hematopoietic stem cell transplant
KID: Kids’ inpatient database
AHRQ: Agency for healthcare research and quality
HCUP: Healthcare resource utilization project
LOS: Length of stay
UCB: Umbilical cord blood
MMUD: Mismatched unrelated donor transplants
MUD: Matched unrelated donors
ER: Emergency room
XLT: X linked thrombocytopenia
XLN: X linked neutropenia
